# Physicochemical Pretreatment of *Vietnamosasa pusilla* for Bioethanol and Xylitol Production

**DOI:** 10.3390/polym15193990

**Published:** 2023-10-04

**Authors:** Suwanan Wongleang, Duangporn Premjet, Siripong Premjet

**Affiliations:** 1 Department of Biology, Faculty of Science, Naresuan University, Muang, Phitsanulok 65000, Thailand; suwananwo64@nu.ac.th; 2 Department of Agricultural Science, Faculty of Agriculture, Natural Resources and Environment, Naresuan University, Muang, Phitsanulok 65000, Thailand

**Keywords:** weed feedstock, biomass hydrolysate, NaOH pretreatment, bioethanol, xylitol

## Abstract

The consumption of fossil fuels has resulted in severe environmental consequences, including greenhouse gas emissions and climate change. Therefore, transitioning to alternative energy sources, such as cellulosic ethanol, is a promising strategy for reducing environmental impacts and promoting sustainable low-carbon energy. *Vietnamosasa pusilla*, an invasive weed, has been recognized as a high potential feedstock for sugar-based biorefineries due to its high total carbohydrate content, including glucan (48.1 ± 0.3%) and xylan (19.2 ± 0.4%). This study aimed to examine the impact of NaOH pretreatment-assisted autoclaving on *V. pusilla* feedstock. The *V. pusilla* enzymatic hydrolysate was used as a substrate for bioethanol and xylitol synthesis. After treating the feedstock with varying concentrations of NaOH at different temperatures, the glucose and xylose recovery yields were substantially higher than those of the untreated material. The hydrolysate generated by enzymatic hydrolysis was fermented into bioethanol using *Saccharomyces cerevisiae* TISTR 5339. The liquid byproduct of ethanol production was utilized by *Candida tropicalis* TISTR 5171 to generate xylitol. The results of this study indicate that the six- and five-carbon sugars of *V. pusilla* biomass have great potential for the production of two value-added products (bioethanol and xylitol).

## 1. Introduction

The depletion of nonrenewable fossil fuel reserves is impending, leading to a severe global energy crisis [1,2]. Consequently, the increasing demand for sustainable and renewable energy sources has driven extensive research into the production of renewable fuels and valuable compounds from nonedible biomass feedstocks [1,2,3,4]. Lignocellulosic biomass is abundant, cost-effective, and may form part of an environmentally friendly conversion process [5]. Extensive research has identified lignocellulose as an alternative energy source because of its high cellulose content, which makes it a suitable bioethanol production substrate [5,6,7,8].

*Vietnamosasa pusilla,* commonly known as Pai Pek (local Thai name), belongs to the Poaceae family and is a drought-resistant weed. *V. pusilla* has been classified as a “regionally controlled weed,” indicating its status as a risk group for a specific region’s primary crops and the environment [9,10]. *V. pusilla* has been documented to be distributed in a geographic range that includes Cambodia, Laos, Thailand, and Vietnam [11]. The major advantage of weed biomass is its rapid development despite harsh environmental conditions and minimal cultivation requirements [12]. Previous research has demonstrated the potential of *V. pusilla* biomass for sugar-based biorefineries to produce bioethanol as a fuel [6]. However, the recalcitrant structure of all lignocellulosic materials, such as hemicellulose, lignin, and cellulose, makes digestion with enzymes difficult, leading to a lower bioconversion yield [3,13,14]. Therefore, pretreatment is crucial for the enhancement of sugar conversion and the facilitation of the release of fermentable sugars [13,14]. Among the various pretreatment methods, alkaline pretreatment has gained wide acceptance because of its simplicity, non-corrosiveness, lack of pollutants, and strong pretreatment effectiveness under mild reaction conditions, leading to fewer inhibitory compounds [15]. This method creates an intricate lignin–hemicellulose network, enhancing lignin removal and increasing the surface area and porosity of the biomass. Consequently, enzymatic hydrolysis is enhanced, and a higher fraction of carbohydrates is preserved [13,14,16]. NaOH pretreatment, which is a common alkaline pretreatment, effectively breaks down lignin by solubilizing lignin carbohydrate bonds, increasing the cellulose surface area, and reducing the sugar content [13,14]. Furthermore, the effectiveness of the NaOH pretreatment can be improved by integrating a heating method that involves subjecting the material to high temperatures and pressures. NaOH-assisted thermal pretreatment is considered a highly efficient method for breaking down the recalcitrant structure of lignocellulose while minimally impacting cellulose and hemicellulose. This process effectively removes a large amount of lignin, reduces cellulose crystallinity, and enhances the specific surface area of cellulose for enzymatic saccharification [13,14,17]. This method has been successfully utilized for the pretreatment of several types of biomasses, including rice straw [18], water hyacinth [19], soybean hull [20], cassava [21,22], *Arachis hypogea* shells [16], sugarcane bagasse [23], *Pennisetum purpureum* [24,25], wheat straw [26], durian peel [17], *Sicyos angulatus* [15], and *Miscanthus × giganteus* [27].

This study aimed to determine the effects of NaOH pretreatment-assisted autoclaving on *V. pusilla* feedstock. The *V. pusilla* enzymatic hydrolysate was used as a substrate for bioethanol and xylitol production. Our findings may help in optimizing the efficiency of cellulosic ethanol and xylitol production using plant-based biomass.

## 2. Materials and Methods

### 2.1. Sample Preparation

The above-ground biomass of *V. pusilla* was collected from a naturally occurring location in the Wang Thong neighborhood of Phitsanulok, Thailand. The specimen was subjected to a cleansing process using tap water and, following that, completed a drying period of five days under shaded conditions. Subsequently, the specimens were separated into shorter pieces of around 5 cm in length and subsequently subjected to pulverization through the utilization of a wood mill (Retsch, Haan, Germany). For subsequent examination and analysis, the powder was sieved through a 150–300 µm laboratory test sieve and stored in bottles at 25 °C.

### 2.2. Analytical Processes

The details of these analytical methodologies are described in a previous report by Premjet et al. [28]. The composition of *V. pusilla* feedstock was investigated before and after alkali pretreatment. National renewable energy laboratory techniques were employed to evaluate the sugar and lignin contents of the sample [29]. Ultraviolet–visible (UV-Vis) spectrophotometer (Analytik Jena Specord 40, Analytik Jena AG, Jena, Germany) absorbance at 205 nm was utilized to assess acid-soluble lignin.

Monomeric sugar, ethanol, and xylitol were analyzed using a high-performance liquid chromatography (HPLC; Agilent 1100, Agilent Technologies, Waldbronn, Germany) system integrated with a refractive index detector (G1362A; Agilent Technologies, Waldbronn, Germany) and a Bio-Rad Aminex-HPX 87H column (300 mm × 7.8 mm; Hercules, CA, USA), which were maintained at 55 and 60 °C, respectively. The mobile phase consisted of 0.005 M sulfuric acid with a 0.6 mL/min flow rate. The injection volume was 20 µL [30].

### 2.3. Alkaline-Catalyzed Steam Pretreatment of V. pusilla Feedstock

The pretreatment technique was employed in a previous study conducted by Obeng et al. [12]. In a 125-mL Erlenmeyer flask, 3 g of *V. pusilla* feedstock was soaked in 24 mL of NaOH solution (1, 2, 3, and 4% *w*/*v*), and the flasks were autoclaved (Hirayama, HVA-85, Toyono-cho, Kasukabe-shi, Saitama, Japan) at 120 °C and 15 psi of pressure for 60 min. Then, the *V. pusilla* sample with the ideal NaOH concentration was subjected to treatment at 110, 120, and 130 °C at 15 psi of pressure for 60 min. At the end of the pretreatment process, the slurry was rapidly ice-cooled and then filtered. The solid portion was washed thoroughly with deionized water to attain a neutral pH. The washed alkaline-catalyzed steam pretreated biomass samples were used for further analysis [12].

Equations (1) and (2) were used to evaluate the recovery yield (%) and lignin removal (%) [12]:

(1) $$Recovery\;yield\left(\%\right)=\frac{Solid\;recovery\;of\;each\;content\left(\%\right)\times Treated\;composition\;of\;each\;content(\%)}{Untreated\;composition\;of\;each\;content(\%)}$$

(2) $$Lignin\;removal\left(\%\right)=100-lignin\;recovery$$

### 2.4. Enzymatic Hydrolysis

The process of enzymatic hydrolysis of both raw and treated *V. pusilla* biomass was monitored using methods that have been previously reported with slight modifications. [12]. A 50 mL Erlenmeyer flask containing 10 mL of digestion solution was used to hydrolyze 0.1 g of biomass (dry weight) from both the raw and pretreated samples. The enzyme mixture used in the digestion process included 30 filter paper units (FPU) of cellulase (*Trichoderma reesei* C2730, Sigma-Aldrich, St. Louis, MO, USA), 60 U xylanase (*Trichoderma longibrachiatum* X2629, Sigma-Aldrich, Co., St. Louis, MO, USA), and 60 U-glucosidase (Oriental Yeast Co., Ltd., Tokyo, Japan) per gram of dry biomass in a sodium citrate buffer (pH 4.8) with 2% (*w*/*v*) sodium azide. The flask of each reaction was incubated at 50 °C and 150 rpm for 96 h in a rotary shaker (Innova 4340, New Brunswick Scientific Company, Edison, NJ, USA). At 12, 24, 48, 72, and 96 h intervals, 200 µL of hydrolysate were collected for sugar determination [12].

The hydrolysis efficiencies of glucose (HEG) and xylose (HEX), glucose recovery (GR), and xylose recovery (XR) were determined using the following equations from Obeng et al. [12] and Sun et al. [5]:

(3) $$HEG\left(\%\right)=\frac{Glucose\;generated\;by\;hydrolysis\left(g\right)\times 100}{1.11*Initial\;glucan\;in\;substrate\;(g)}$$

(4) $$HEX(\%)=\frac{Xylose\;generated\;by\;hydrolysis\left(g\right)\times 100}{1.14*Initial\;amount\;of\;xylan\;in\;substrate\;(g)}$$

(5) $$GR\left(\%\right)=\left(solid\;recovery\times glucan\;in\;substrate\times 1.11\times HEG\right)\times 100$$

(6) XR(%)=(solid recovery×xylan in substrate×1.14×HEX)×100

where 1.11 and 1.14 are the conversion factors for glucan and xylan to glucose and xylose, respectively.

### 2.5. Structure and Crystallinity Analysis of Feedstock

The analytical techniques had been followed in a previous investigation [12,17]. The raw and treated *V. pusilla* feedstocks were freeze-dried and fixed on aluminum stumps using double-sided carbon tape and a gold coating. A field emission scanning electron microscope (FESEM; Thermo Fisher Scientific, Apero S, Waltham, MA, USA) was used to observe modifications in the surface morphology of all specimens [12,17].

Changes in the crystallinity index (*CrI*) of raw and treated samples were determined by X-ray diffraction (XRD) using a PANalytical X’pert Pro, PW 3040/60 diffractometer (Almelo, The Netherlands). The biomass samples underwent a triple wash with acetone and were thereafter subjected to drying at a temperature of 32 °C. The dry specimens were pulverized to achieve a particle size that passed through a 150 µm mesh screen. The diffraction intensity was determined within the angular range of 2θ = 10–40°, using a scanning rate of 0.02° s^−1^/min [12,17].

Using the Segal equation, the *CrI* was determined as follows [31]:

(7) $$CrI=(I_{002}-I_{am})∕I_{002}\times 100\%$$

where *I_am_* is the lowest intensity of the amorphous sector at 2θ = 18.0° and *I*_002_ is the greatest intensity of the crystallinity sector at 2θ = 22.0°.

### 2.6. Microorganisms

In this study, bioethanol and xylitol were produced using *Saccharomycis cerevisiae* TISTR 5339 and *Candida tropicalis* TISTR 5171, respectively. These yeast strains were purchased in special order and submitted to the Thailand Institute of Science and Technology (TISTR).

### 2.7. Preparation of Seed Culture

A single loop from each yeast strain, *S. cerevisiae* TISTR 5339 and *C. tropicalis* TISTR 5171, was used as the initial inoculum. *S. cerevisiae* TISTR 5339 was placed into a 50 mL Falcon tube with 10 mL of yeast malt medium and incubated with a rotating shaker (Innova 4340, New Brunswick Scientific Company, Edison, NJ, USA) at 30 °C for 18 h. *C. tropicalis* TISTR 5171 was transferred to a 50 mL Falcon tube containing 10 mL of yeast malt peptone medium and incubated using a rotary shaker at 30 °C for 24 h.

### 2.8. Preparing Biomass Hydrolysate for Ethanol Production

The biomass hydrolysate (BH) was prepared according to procedures outlined in a previous study [32]. After the *V. pusilla* biomass was treated under optimal conditions, followed by enzymatic saccharification, the enzymatic activity was terminated by heating the BH to 100 °C in a water bath (Grant Instrument, Grant W28, Ltd. Barrington, Cambridge, UK) for 20 min. The solid components of the biomass were separated by centrifugation at 12,000× *g* for 2 h before being filtered through a glass microfiber filter (Whatman, Amersham Place, Little Chalfont, Buckinghamshire HP7 9NA, UK). The BH was then concentrated using a rotary vacuum evaporator (Heidolph Instruments, Hei-VAP Advantage, GmbH & Co. KG Walpersdorfer Str., Schwabach, Germany) until it yielded approximately 20 g/L glucose. The pH of the BH was adjusted to 6 using a 1 M NaOH solution and chilling overnight at 4 °C. Subsequently, the mixture was centrifuged for 2 h at 12,000× *g* and passed through a glass filter. The BH was subsequently maintained at 4 °C for use in future experiments [32].

### 2.9. Preparing Biomass Hydrolysate for Xylitol Production

The liquid fraction, a byproduct of ethanol production, was separated by centrifugation at 12,000× *g* for 1 h and passed through a glass microfiber filter to remove yeast cells and other contaminants. Subsequently, a rotary vacuum evaporator containing 20 g/L xylose was used to condense the liquid into xylose biomass hydrolysate (XBH). The pH of XBH was adjusted to 5.5 and stored overnight at 4 °C. Then, the mixture was centrifuged for 1 h at 12,000× *g* and passed through a glass filter. The XBH was then stored at 4 °C for use in future studies [32].

### 2.10. Medium for Bioethanol and Xylitol Production

Ethanol was produced using the control and the BH media. The BH medium contained glucose derived from the biomass, whereas commercial glucose was used as the carbon source in the control medium. Each medium was prepared by adding 20 g/L glucose, 10 g/L peptone, 10 g/L yeast extract, 2 g/L MgSO_4_, and 2 g/L K_2_HPO_4_, and the pH was set at 6 [32].

Xylitol production requires XBH and control media. The XBH medium used xylose derived from biomass as its carbon source, whereas the control medium relied on commercial xylose. Each medium contained 20 g/L xylose, 5 g/L yeast extract, 1 g/L MgSO_4_, 1 g/L KH_2_PO_4_, and 1 g/L NH_4_Cl at a pH of 5.5 [33].

In this study, all liquid culture media were prepared under aseptic conditions and passed through a Millipore filter with a 0.2 µm pore size (Merck Ltd. Tullagreen, Carrigtwohill, Co., Cork, UK).

### 2.11. Ethanol Fermentation

The specifications of the bioethanol production methodology were followed in a previous study by Premjet, et al. [32]. The production of bioethanol utilized 50 mL of both BH and a control medium. Both media were inoculated with 2% (*v*/*v*) *S. cerevisiae* TISTR 5339 seed culture and incubated at 30 °C on a rotary shaker (Innova 4340, New Brunswick Scientific Company, Edison, NJ, USA) at 150 rpm for 24 h. A milliliter of the liquid fraction was collected for HPLC analysis after 3, 6, 9, 12, 15, 18, 21, and 24 h of incubation to assess glucose consumption and ethanol production [32]. The ethanol yield was determined using the following equation:

(8) $$Ethanol\;yield\;(\%)=\frac{Ethanol\;in\;fermentation\;liquid\left(g\right)\times 100}{0.511\times Glucose\;initial\;(g)}$$

### 2.12. Xylitol Fermentation

The total volume of the XBH and control media used for xylitol production was 50 mL. A 5% seed culture of *C. tropicalis* TISTR 5171 was injected into both media and then incubated on a rotary shaker (Innova 4340, New Brunswick Scientific Company, Edison, NJ, USA) at 200 rpm for 7 days at 30 °C. The amounts of xylose consumed, and xylitol produced, were subsequently determined by collecting 1 mL of the liquid fraction daily for HPLC analysis. The xylitol yield was determined using the following equation:

(9) $$Xylitol\;yield\;(\%)=\frac{Xylitol\;in\;fermentation\;liquid\left(g\right)\times 100}{0.905\times Xylose\;initial\;(g)}$$

### 2.13. Determination Growth of Microorganisms

The optical density at 600 nm was determined using a UV spectrophotometer to monitor the yeast cell growth [32]. At 600 nm, an OD of 1.0 corresponds to approximately 1.5 × 10^7^ cells/mL.

## 3. Results

### 3.1. Composition of Raw Biomass

The chemical structure of the *V. pusilla* biomass (dry weight) sample comprised glucan (48.1 ± 0.3%), xylan (19.2 ± 0.4%), arabinan (1.2 ± 0.1%), acid insoluble lignin (AIL: 23.5 ± 0.1%), and acid soluble lignin (ASL: 4.4 ± 0.1%). These components constituted approximately 68.5 and 27.9% of the total carbohydrate and lignin contents, respectively. In a previous study, this composition was evaluated and discussed [6].

### 3.2. Effect of NaOH Concentration on Composition

*V. pusilla* feedstock was pretreated with 1, 2, 3, and 4% NaOH at 120 °C for 60 min to determine the optimal NaOH concentration for pretreatment. Changes in the chemical composition of the biomass before and after pretreatment with varying concentrations of NaOH are presented in Table 1. This study revealed that NaOH pretreatment significantly influences the partial dissolution of lignin and carbohydrates from this weed biomass.

As the concentration of NaOH increased, both glucan and xylan contents increased significantly (*p* < 0.05). The relative glucan content gradually increased by 55.3 ± 0.5, 67.2 ± 0.1, 68.0 ± 0.0, and 71.0 ± 0.2%, when treated with 1, 2, 3, and 4% NaOH, respectively. The xylan content increased by 21.9 ± 0.2 and 21.3 ± 0.3% after treatment with 1 and 2% NaOH, respectively, although the improvements were not statistically significant (*p* < 0.05). When the concentration of NaOH increased to 3 and 4%, the xylan content declined substantially to 20.1 ± 0.2 and 17.8 ± 0.4%, respectively. Arabinan was not detected in any treated samples (Table 1).

**Figure 1:**
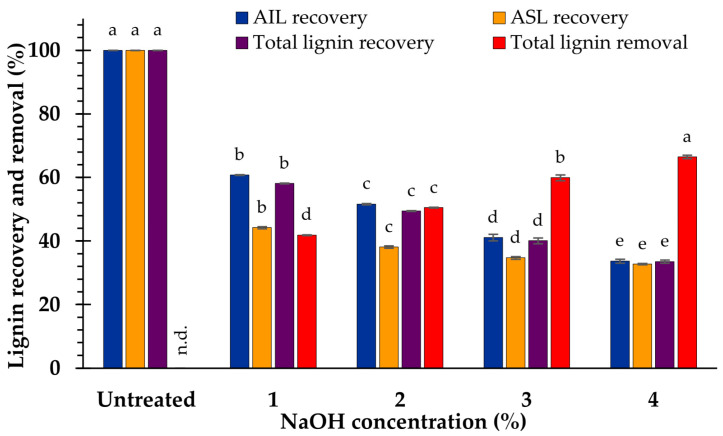
Lignin recovery and removal after pretreatment at difference NaOH concentration. The significance of the variations in concentrations (*p* < 0.05), is shown by the superscripted characters; n.d. = not determined.

In addition, increasing the NaOH concentration from 1 to 4% resulted in substantial declines in AIL and total lignin content. The minimum quantities of AIL (15.4 ± 0.3%) and the total lignin (18.3 ± 0.3%) were obtained by feedstock treated with 4% NaOH. However, ASL content significantly decreased (*p* < 0.05) with 1% NaOH, whereas higher concentrations of NaOH (2–4%) had no effect on ASL reduction (Table 1). The results reveal that the total lignin content declined from 27.9 ± 0.2 (raw biomass) to 18.3 ± 0.3%. Approximately 66% of delignification was attributed to this pretreatment method (Figure 1).

**Figure 2:**
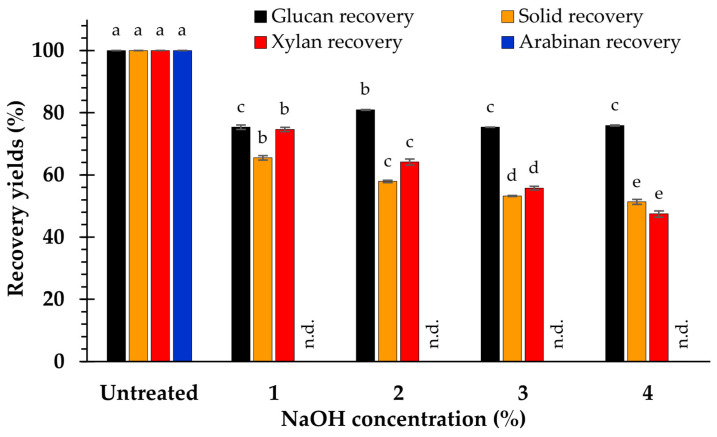
Recovery yields after pretreatment at difference NaOH concentration. The significance of the variations in concentrations (*p* < 0.05), is shown by the superscripted characters; n.d. = not determined.

The results reveal a gradual reduction in the recovery yields of solids, xylan and glucan, which progressively declined as the NaOH concentration raised (Figure 2). After treating the feedstock with 1–4% NaOH, the solid recovery yields ranged from 51.4 ± 0.8 to 65.5 ± 0.7%, whereas the xylan recovery yields ranged from 47.5 ± 1.0 to 74.7 ± 0.6%. However, there was no significant (*p* < 0.05) difference in the glucan recovery yield after pretreatment with 1, 3, or 4% NaOH. NaOH concentrations of 2% resulted in the highest glucan recovery yields (81.0 ± 0.1%). The results indicate that, by pretreating the sample with 1–4% NaOH, solid and glucan recovery yields greater than 50 and 75% were achieved, respectively. Nevertheless, a xylan recovery yield greater than 50% was obtained by treatment with 1–3% NaOH. The maximum yields for solid recovery (65.5 ± 0.7%) and xylan recovery (74.7 ± 0.6%) were achieved with 1% NaOH, whereas the highest yield for glucose recovery (81.0 ± 0.1%) was acquired with 2% NaOH.

### 3.3. Effect of NaOH Concentration on Hydrolysis Yield

To determine the suitable NaOH concentration for the pretreatment of weedy feedstock, the treated biomass samples were subjected to enzymatic hydrolysis. The highest yields of GR, HEG, XR, and HEX were obtained after 96 h of hydrolysis. The findings reveal that the yields of untreated feedstock for GR, HEG, XR, and HEX were 11.5 ± 0.0, 19.4 ± 0.0, 5.0 ± 0.0, and 20.1 ± 0.1%, respectively. After treating the feedstock with 1, 2, 3, and 4% NaOH, the yields of GR, HEG, XR, and HEX were significantly (*p* < 0.05) higher than those of the untreated material. However, the increase in HEG yields of 2 (88.4 ± 0.0%), 3 (87.8 ± 0.2%), and 4% NaOH (87.7 ± 0.3%) did not differ statistically. Nevertheless, the greatest yield of GR (42.4 ± 0.0%) was obtained from a sample treated with 2% NaOH.

**Figure 3:**
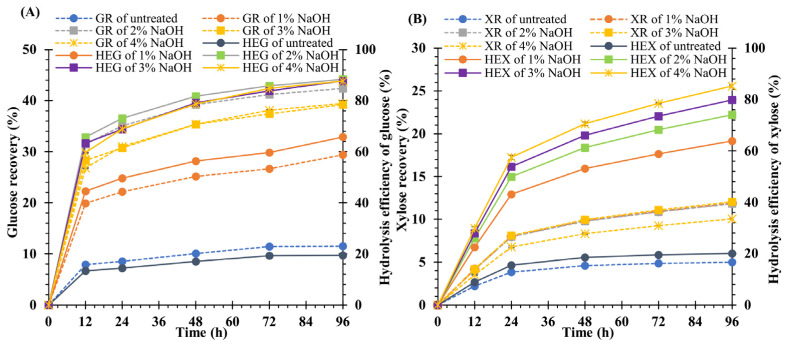
(**A**) Glucose recovery (GR) and hydrolysis efficiency of glucose (HEG) and (**B**) xylose recovery (XR) and hydrolysis efficiency of xylose (HEX) of untreated and pretreated biomass of *V. pusilla.*

Moreover, the levels of GR diminished at NaOH concentrations of 3 (39.2 ± 0.1%) and 4% (39.5 ± 0.1%), but no significant differences were observed (*p* < 0.05). The highest yield of HEX (85.3 ± 0.4%) was generated from the sample treated with 4% NaOH, whereas the yield of XR (10.1 ± 0.0%) was the lowest. However, the XR yields were enhanced at 1 (11.9 ± 0.1), 2 (11.8 ± 0.1), and 3% (12.1 ± 0.1) NaOH, but the difference was not statistically significant between them (Figure 3A,B). Additionally, treatment of *V. pusilla* biomass with 2% NaOH at 120 °C for 60 min resulted in 3.7- and 2.4-fold boosts in GR and XR yields, respectively, when compared with untreated feedstock. The yields of HEG and HEX increased by approximately 4.6 and 3.7 times, respectively.

### 3.4. Effect of Temperature on Composition

To determine the suitable pretreatment temperature, *V. pusilla* feedstock was treated with 2% NaOH for 60 min at various temperatures. The relative glucan content increased to 65.1 ± 0.3, 67.2 ± 0.1, and 67.1 ± 0.3% when the temperature was raised to 110, 120, and 130 °C, respectively. The increase in glucan content at 120 and 130 °C was not statistically different (*p* < 0.05). However, the maximum relative xylan content (22.5 ± 0.1%) was observed at 110 °C, which was subsequently reduced to 21.3 ± 0.3 and 19.3 ± 0.2% at 120 and 130 °C, respectively. Arabinan did not appear in any of the treatment materials (Table 2). 

**Figure 4:**
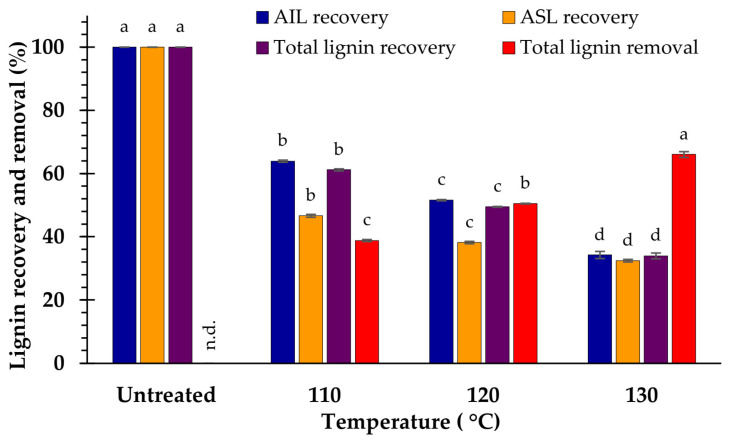
Lignin recovery and removal after pretreatment at different temperatures. The significance of the variations in temperature (*p* < 0.05), is shown by the superscripted characters; n.d. = not determined.

At 110 °C, a slight variation in AIL content between the treated (23.0 ± 0.1%) and untreated samples (23.5 ± 0.1%) was observed. The AIL content was then substantially reduced at higher temperatures (120 and 130 °C). Moreover, a decrease in ASL was observed when the temperature increased. The amount of ASL diminished at 120 (2.9 ± 0.0%) and 130 °C (2.9 ± 0.0%) and did not differ significantly (*p* < 0.05). Nevertheless, a gradual decrease in the total lignin content coupled with an increase in the pretreatment temperature was observed. Feedstock treated at 130 °C resulted in the smallest amounts of AIL (16.1 ± 0.5%), ASL (2.9 ± 0.0%), and total lignin (19.0 ± 0.5%). The evidence showed that the overall amount of lignin decreased from 27.9 ± 0.2 (untreated sample) to 19.0 ± 0.5% (at 130 °C). This pretreatment strategy was responsible for approximately 66% delignification, which was equivalent to that of the sample treated with 4% NaOH, as shown in Figure 1 and Figure 4.

**Figure 5:**
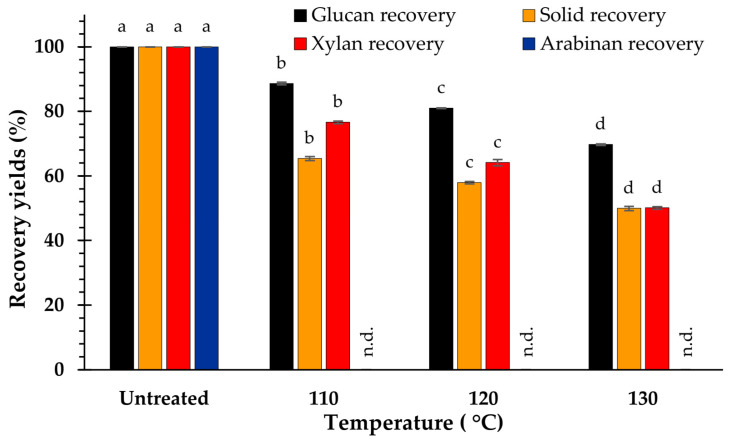
Recovery yields after pretreatment at different temperatures. The significance of the variations in temperature (*p* < 0.05), is shown by the superscripted characters; n.d. = not determined.

The results reveal that the temperature increase accompanied a reduction in the solid, xylan, and glucan recovery yields. At 110 °C, the highest recovery yields were obtained for solids (65.4% ± 0.6%), glucan (88.6% ± 0.4%), and xylan (76.6% ± 0.4%). However, the minimum amounts of the solid (49.9 ± 0.7%), xylan (50.1 ± 0.6%), and glucan (69.7 ± 0.3%) recovery yields were obtained at 130 °C. Therefore, the temperature may significantly influence the decline in the recovery yields of solids, xylan, glucan, and total lignin (Figure 5).

### 3.5. Effect of Temperature on Hydrolysis Yield

**Figure 6:**
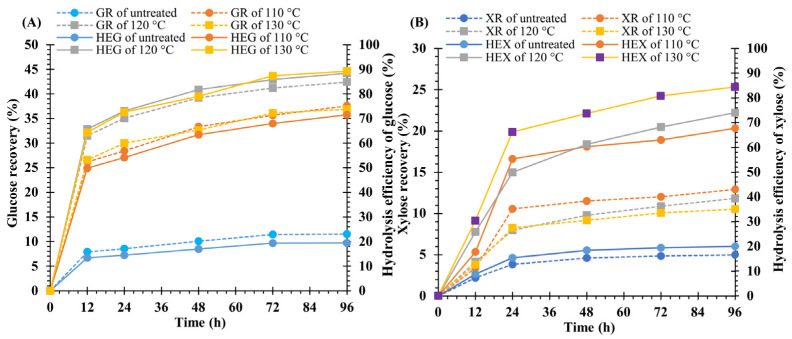
(**A**) Glucose recovery (GR) and hydrolysis efficiency of glucose (HEG) and (**B**) xylose recovery (XR) and hydrolysis efficiency of xylose (HEX) of untreated and pretreated biomass of *V. pusilla.*

To determine the optimal pretreatment temperature, the biomass of *V. pusilla* was treated with 2% NaOH at various temperatures and subjected to enzymatic hydrolysis. Pretreatment of the feedstock with 2% NaOH at 110, 120, and 130 °C was demonstrated to considerably increase the quantities of GR, HEG, XR, and HEX (Figure 6A,B). Compared with the untreated sample, the GR yields expanded to 37.6 ± 0.1, 42.4 ± 0.0, and 36.9 ± 0.1% at 110, 120, and 130 °C, respectively. When the temperature increased, the HEG and HEX yields increased steadily. The highest yields of HEG (89.3 ± 0.3%) and HEX (84.4 ± 0.2%) were achieved at 130 °C. Whereas the highest yield of XR (12.9 ± 0.0%) was achieved at 110 °C. The yields dropped dramatically at higher temperatures (120 and 130 °C). These findings confirm that samples treated with 2% NaOH at 120 °C for 60 min produced the highest yield of GR (42.4 ± 0.0%).

### 3.6. Effect of Pretreatment on Crystalline Structure

**Figure 7:**
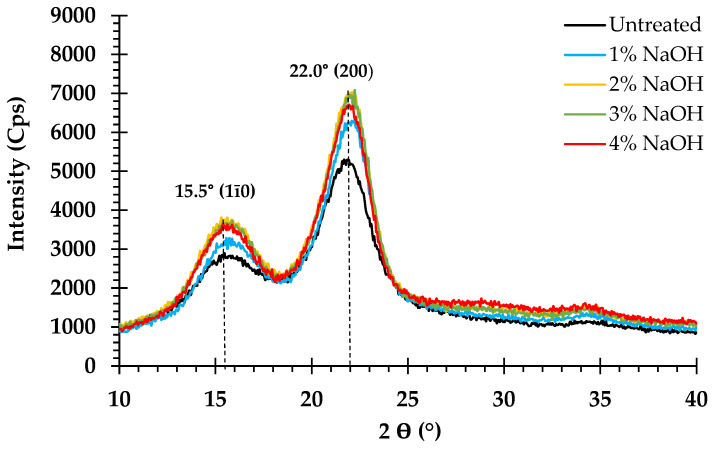
X-ray diffraction pattern of untreated and pretreated *V. pusilla* with varying concentrations of NaOH.

**Figure 8:**
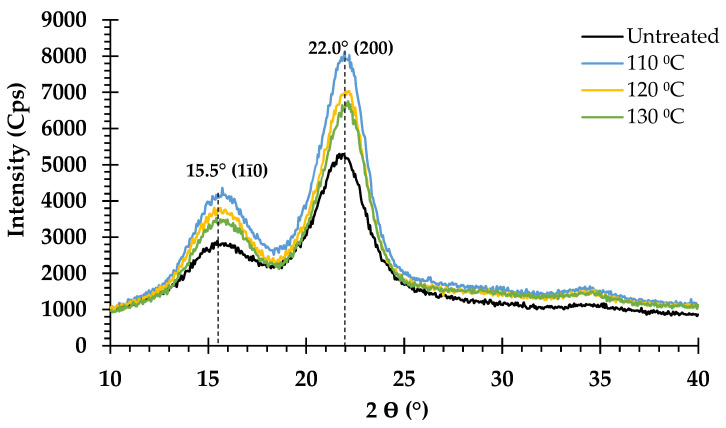
X-ray diffraction pattern of untreated and 2% NaOH treated *V. pusilla* with varying temperatures.

The effect of NaOH pretreatment on *V. pusilla* crystalline cellulose was investigated using XRD. Consequently, all the untreated and treated biomass samples with varying NaOH concentrations and temperatures were analyzed. The XRD patterns are shown in Figure 7 and Figure 8. All the treated and untreated samples were identified as cellulose I based on their XRD patterns, which consisted of two significant peaks at 2θ = 15.5° and 2θ = 22°. The untreated sample exhibited the lowest *CrI* value (59.5%). The *CrI* values of all treated samples increased after pretreatment with different NaOH concentrations and temperatures applied to the feedstock. The sample treated with 1–4% NaOH exhibited increased *CrI* values ranging from 66.2 to 67.9%. However, when this sample was treated at various temperatures, the *CrI* increased from 67.4 to 68.5% (Table 3). 

### 3.7. Effect on Morphological Structure

**Figure 9:**
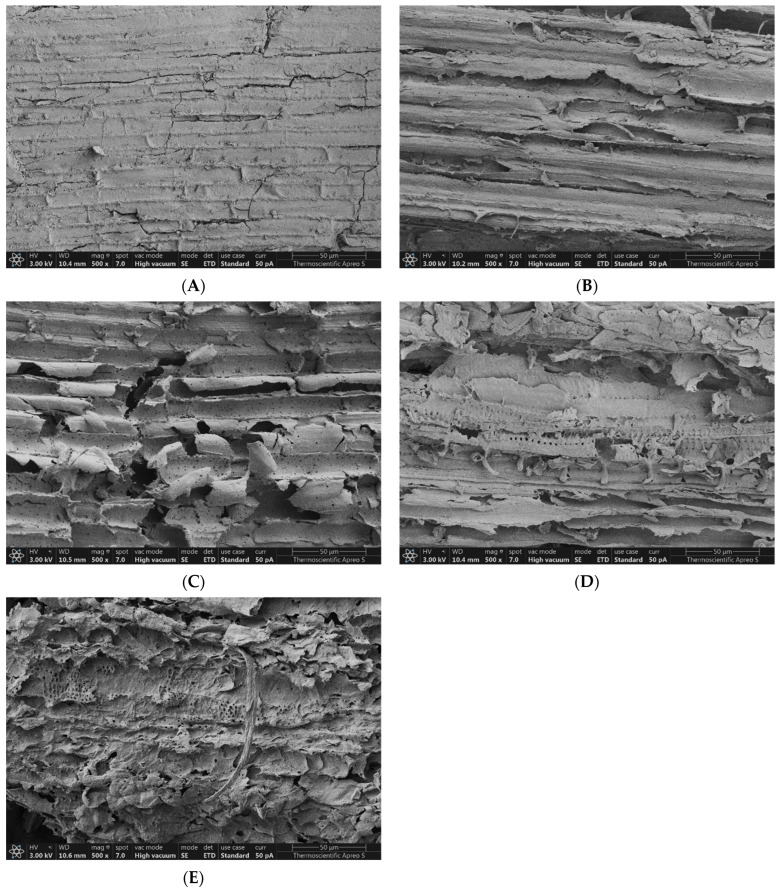
Surface morphology of (**A**) untreated and NaOH-pretreated samples at (**B**) 1, (**C**) 2, (**D**) 3, and (**E**) 4% NaOH.

**Figure 10:**
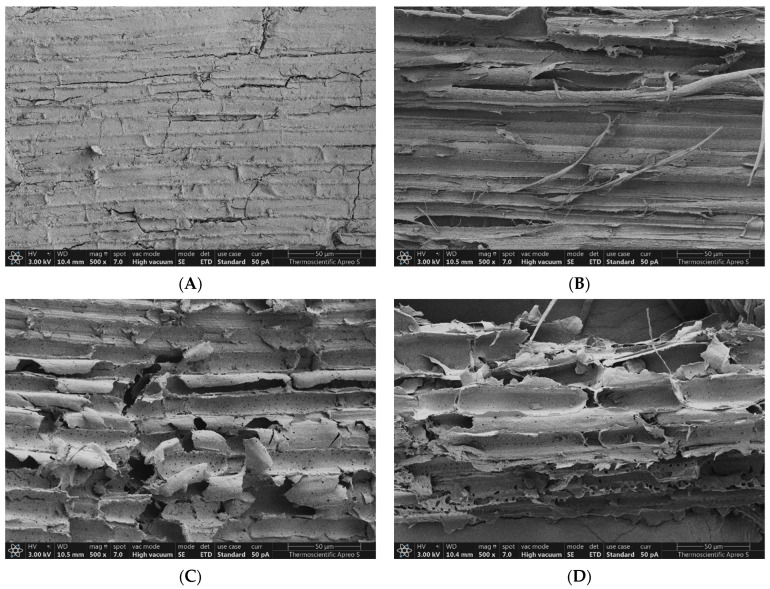
Surface morphology of (**A**) untreated and 2% NaOH-pretreated samples at (**B**) 110, (**C**) 120, and (**D**) 130 °C.

SEM was used to evaluate the surface morphology of untreated *V. pusilla*, which was subjected to alkaline-assisted thermal pretreatment with varying concentrations of NaOH and temperatures. The results are shown in Figure 9A–E and Figure 10A–D. The untreated specimens exhibited a dense morphology characterized by bundled fiber organization and a smooth surface devoid of porosity. Following exposure to different concentrations of NaOH and temperatures, the surface morphology of the sample was considerably altered, resulting in the disorganization of the fibers. Furthermore, an increase in NaOH concentration and temperature resulted in a higher degree of surface fragmentation, which was characterized by the presence of distinct elongated fissures, deep crevices, and pores (Figure 9A–E and Figure 10A–D).

### 3.8. Ethanol Fermentation

**Figure 11:**
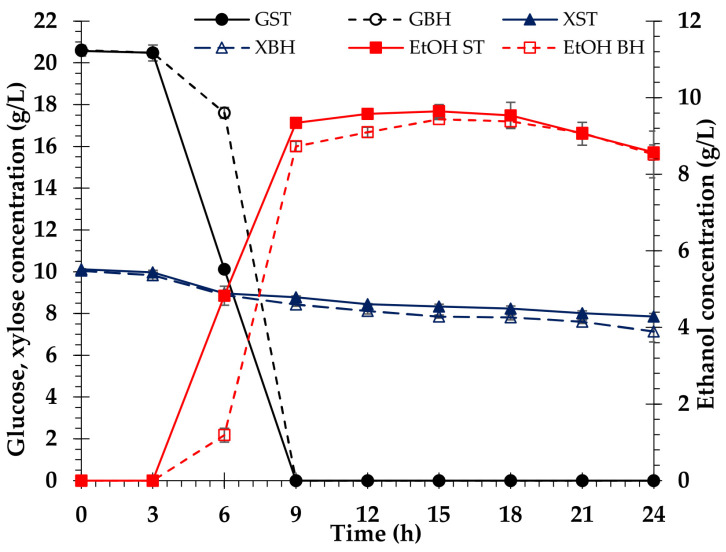
Profiles of sugar consumption and bioethanol production of *S. cerevisiae* TISTR 5339 in standard and biomass hydrolysate. Glucose consumption in standard (GST) medium and glucose consumption in biomass hydrolysate (GBH) medium. Xylose consumption in standard (XTS) medium and xylose consumption in biomass hydrolysate (XBH) medium. Ethanol in standard (EtOH ST) medium and biomass hydrolysate (EtOH BH) medium.

**Figure 12:**
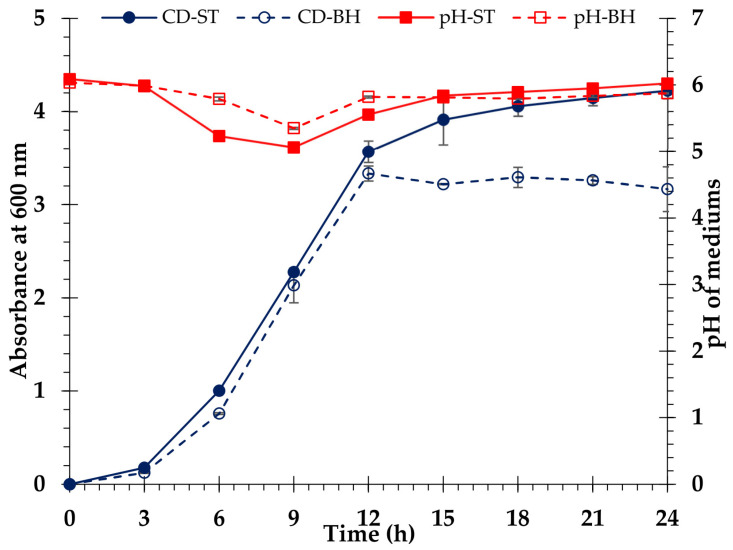
Cell density of *S. cerevisiae* TISTR 5339 in standard (CD-ST) medium and biomass hydrolysate (CD-BH) medium. The pH in standard (pH-ST) medium and biomass hydrolysate (pH-BH) medium.

Enzymatic hydrolysis produces a sugar solution called BH, which contains glucose and xylose. To utilize the glucose in *V. pusilla* BH for ethanol fermentation, the BH was concentrated using a rotary vacuum evaporator to generate approximately 20 g/L glucose. Upon completion of the concentration procedure, BH was found to contain a mixture of 20 and 10 g/L of glucose and xylose, respectively. These findings reveal that xylose, a byproduct of BH, has great potential for utilization in the production of other high-value products. To obtain the maximum benefit from *V. pusilla* BH medium, we first fermented the medium without detoxification using *S. cerevisiae* TISTR 5339 to create bioethanol. Within 24 h, we analyzed the consumption of glucose and xylose, amount of bioethanol, exponential growth of yeast cells, and pH of the media to evaluate the potential of *V. pusilla* BH medium for bioethanol production. The standard medium contained 20 g/L of D-glucose (98% reagent grade) and 10 g/L of xylose (99% reagent grade). The results are summarized in Figure 11 and Figure 12. The yeast strain *S. cerevisiae* TISTR 5339 rapidly consumes glucose and concurrently produces ethanol. In both standard and BH media, glucose was consumed entirely within 9 h. After 15 h of incubation, the standard medium generated a maximal ethanol yield of 91.8 ± 1.6% or 9.7 ± 0.2 g/L, whereas the BH medium allowed a yield of 89.6 ± 0.1% or 9.4 ± 0.0 g/L. After incubation for 15 h, ethanol production in both media decreased slightly. Additionally, xylose in both media was slightly diminished, but it was still present in the standard (7.9 ± 0.2 g/L) and the BH media (7.1 ± 0.5 g/L), as shown in Figure 11.

The growth patterns of *S. cerevisiae* TISTR 5339 were similar in both media, and the pH changes were also comparable. After 24 h of incubation, the yeast growth rate in the standard medium reached its maximum value. However, the highest growth rate of yeast cells was observed after 12 h of incubation in BH medium, with the growth rate decreasing as the incubation duration increased (Figure 12).

### 3.9. Xylitol Production

**Figure 13:**
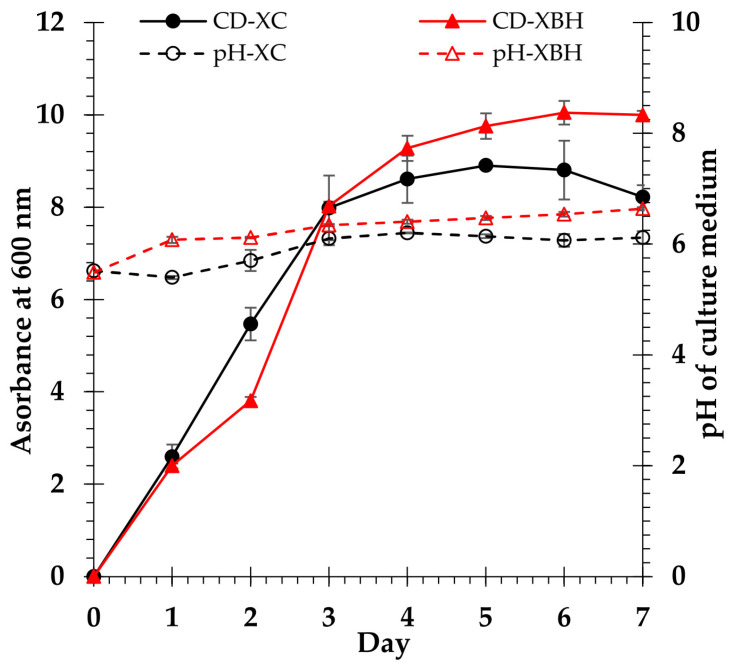
Cell density of *C. tropicalis* TISTR 5171 in xylose control (CD-XC) medium and xylose biomass hydrolysate (CD-XBH) medium. The pH in xylose control (pH-XC) medium and xylose biomass hydrolysate (pH-XBH) medium.

**Figure 14:**
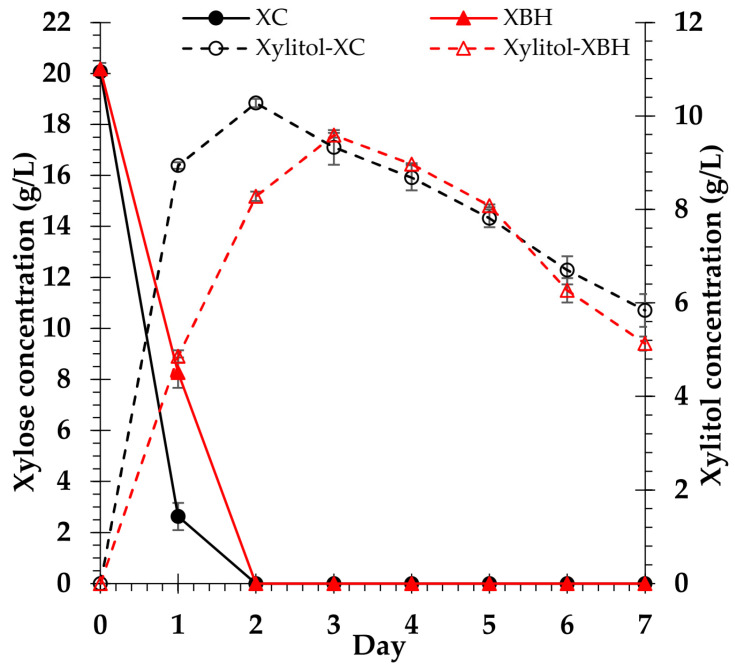
Profiles of xylose consumption and xylitol production of *C. tropicalis* TISTR 5171 in control and biomass hydrolysate media. Xylose consumption in control (XC) medium and xylose consumption in biomass hydrolysate (XBH) medium. Xylitol in control (Xylitol-XC) medium and biomass hydrolysate (Xylitol-XBH) medium.

Following ethanol production, the liquid component was precipitated using centrifugation and filtered to remove yeast cells and a variety of impurities. Subsequently, the liquid was concentrated to a xylose concentration of 20 g/L. These concentrations were then used as carbon sources in the XBH medium for xylitol production. The xylose control (XC) medium used for the evaluation was prepared using D-xylose (99% reagent grade) at a concentration of 20 g/L. *C. tropicalis* TISTR 5171 was cultured in XBH medium without detoxification and XC medium to produce xylitol. Several parameters, including xylose consumption, xylitol concentration, yeast cell proliferation, and medium pH, were examined to evaluate the xylitol-producing potential of each medium. The results of these assessments are shown in Figure 13 and Figure 14. 

During the incubation period, the growth patterns of *C. tropicalis* TISTR 5171 and the alterations in pH observed in both XC and XBH media were comparable. After 5 days of incubation, the yeast reached its maximal growth rate in both XC and XBH media, whereas the pH varied from 5.5 to 6.1 and 5.5 to 6.6 in XC and XBH media, respectively (Figure 13).

The results demonstrate that, within 2 days of incubation, *C. tropicalis* TISTR 5171 entirely consumed xylose in both XC and XBH media, whereas xylitol synthesis steadily increased. Maximum xylitol yield (10.3 ± 0.1 g/L, or xylitol yield 56.6 ± 0.1%) was achieved from the XC medium after 2 days of incubation. However, the highest production of xylitol (9.6 ± 0.1 g/L, or xylitol yield 52.5 ± 0.3%) was achieved after 3 days of culture in the XBH medium. Subsequently, xylitol production steadily declined in both the XC and XBH media (Figure 14).

## 4. Discussion

### 4.1. Effect of NaOH Concentration on Biomass Composition 

Utilizing lignocellulosic materials in sugar-based biorefinery platforms necessitates pretreatment processes to facilitate the breakdown of the recalcitrant structures of plant cell walls [6,34]. Pretreatment of lignocellulosic biomass with NaOH effectively removed lignin and hemicellulose, while only partially solubilizing cellulose, depending on the reaction concentration, duration, and environmental conditions [35].

An increase in relative glucan content can be attributed to the partial elimination of lignin and hemicellulose fractions from the sample, as observed in *Chloris barbata* [12], two varieties of durian peel [17], cassava stem waste [21], *Sida acuta* [32], spent coffee grounds [36], rice straw [37], and *Sicyos angulatus* [15]. According to our findings, the relative xylan content improved markedly with 1–3% NaOH, whereas the xylan content declined significantly (*p* < 0.05) with 4% NaOH, which may be because of the increased breakdown of hemicellulose compounds in the presence of a higher concentration of NaOH [36,38]. At mild concentrations, NaOH (0–3%) had a minor impact on the decomposition of hemicellulose during hydrothermal treatment [39], which resulted in an increase in the relative xylan content. Therefore, the increase in xylan content after NaOH pretreatment is contingent on the lignocellulosic biomass type. This phenomenon has been observed in *Sida acuta* [32], spent coffee grounds [36], rice straw [37], herbaceous and woody lignocelluloses [40], corn cob and sweet sorghum bagasse [41], and *Sicyos angulatus* [15].

Delignification, which is total lignin removal (AIL and ASL) in the pretreatment process, is caused by the OH^˗^ liberated from NaOH, reacting and destroying internal-molecular α- and β-aryl ether and C-C bonds in lignin molecules. Consequently, increasing NaOH concentrations, which resulted in a greater amount of OH^−^, had substantial impacts on lignin degradation [27,42,43,44,45], as demonstrated by a variety of lignocellulosic materials, including brewer’s spent grain [46], wheat straw [47], grass waste [38], and *Pennisetum purpureum* [24]. Moreover, delignification efficiencies in this study ranged from 41.9 ± 0.1 to 66.4 ± 0.5%, which is lower than the pretreatment of *V. pusilla* biomass with H_3_PO_4_ (54.4 ± 1.1 to 82.4 ± 1.2%) [6].

However, the decrease in the recovery yields of solids, xylan, and glucan can be attributed to the impact of NaOH during the weed biomass pretreatment process. This is mainly because NaOH can destroy the covalent bonds between lignin and hemicellulose within the lignin–carbohydrate complex, leading to the disintegration of both lignin and hemicellulose [27,42,43,44,45]. In particular, the disintegration of the H bonds in the OHֿ groups of carbohydrates, including glucan, xylan, mannan, arabinan, and galactan, contributes to the degradation of these sugars [42,44]. Consequently, increasing the NaOH concentration led to greater carbohydrate degradation, which considerably reduced the recovery yields of all solids, glucan, and xylan, as observed in *Chloris barbata* [12], grass waste [38], and durian peel [17]. Additionally, the elimination of lignin and other chemical compounds from the feedstock had a substantial effect on the decreased amount of solid recovery at various NaOH concentrations [12,24], which also occurred in *Sicyos angulatus* [15], cassava stems [21], *Pennisetum purpureum* [24,25], *Sida acuta* [32], grass waste [38], and rice straw [39].

### 4.2. Effect of NaOH Concentration on Hydrolysis Yield

The untreated samples generated the lowest quantity of enzymatic hydrolysis products. Lignocellulosic matter is resistant to decomposition by microorganisms and enzymes because of its rigidity and inherent recalcitrance [48,49,50]. After treating the feedstock with various NaOH concentrations, the yields of GR, HEG, XR, and HEX significantly increased for each treated biomass based on the NaOH concentration. NaOH pretreatment improves the enzymatic hydrolysis yield of this biomass by breaking down the *V. pusilla* feedstock’s complex structure, removing lignin and a portion of the hemicellulose that functions as a physical obstacle during hydrolysis, making carbohydrates more accessible to enzymes [14,48,49,51].

According to the results, 2% NaOH generated the highest GR and XR yields, whereas higher NaOH concentrations resulted in lower GR and XR yields. In contrast, NaOH concentrations of 3–4% produced a higher yield of HEX but had no effect on the yield of HEG. The results indicate that increasing the NaOH concentration accelerated the dissolution of lignin, hemicellulose, and cellulose, resulting in a lower solid recovery yield. A reduction in solid recovery yield has a substantial influence on sugar recovery yield [12,17,19,25,32].

### 4.3. Effect of Temperature on Biomass Composition

The results show that the three main components of the treated samples changed considerably depending on the pretreatment temperature. Decreases in AIL, ASL, and total lignin content were also significantly (*p* < 0.05) influenced by the temperature. However, the pretreatment temperature had distinct effects on the reduction in AIL and ASL. Therefore, subjecting the feedstock to high-temperature NaOH pretreatment can disrupt the complex structure of *V. pusilla* feedstocks by eliminating both lignin and hemicellulose components [52].

Compared with the raw material, the relative glucan content increased with increasing temperature because lignin was mostly removed at higher temperatures [12,17]. However, the hydrothermal pretreatment of biomass with NaOH (1–3%) did not have a substantial impact on hemicellulose component [39], resulting in a maximum increase in the xylan content at 110 °C and reduction at 120 and 130 °C because of the increased degradation of hemicellulose. In addition, as the temperature increased, the recovery yields of particulates, xylan, and glucan decreased drastically, whereas lignin removal was considerably enhanced (Figure 4 and Figure 5). Therefore, higher temperatures accelerate the dissolution of the solid fraction, lignin, and carbohydrates [7,12,17,53,54].

### 4.4. Effect of Temperature on Hydrolysis Yield

Furthermore, the yields of GR, HEG, XR, and HEX were substantially enhanced when the biomass of *V. pusilla* was treated at various temperatures. Compared with other pretreatment temperatures, the yields of GR and XR were lowest at 130 °C. However, HEG and HEX yields were highest at high temperatures, which contributed to a more severe decomposition of carbohydrates, resulting in a decline in the enzymatic saccharification yields of glucose, xylose, and reducing sugars [12,17,19]. The highest amount of GR and an average quantity of XR were obtained at 120 °C. In addition, glucose had a higher recovery yield than xylose, indicating that glucose is the key product of the saccharification process and xylose is a byproduct.

The results demonstrate that both the concentration of NaOH and temperature used in the present study had a substantial effect on the alteration of all three major chemical constituents of the *V. pusilla* feedstock. Although the highest amounts of HEG and HEX were obtained when the samples were pretreated with 4% NaOH at 120 °C and 2% NaOH at 130 °C, these may not be the optimal pretreatment conditions for *V. pusilla* biomass. The optimal pretreatment conditions for such biomass should generate the highest potential GR yields, which are directly proportional to the initial glucan content of the biomass [6,55]. Therefore, the optimal pretreatment of *V. pusilla* biomass in this study was 2% NaOH at 120 °C for 60 min, which produced the highest amount of GR. At this condition, 50.5 ± 0.1% of the lignin was removed, indicating that complete lignin elimination may not be necessary. Compared with the untreated feedstock, 2% NaOH treatment of *V. pusilla* biomass increased the GR and XR yields by approximately 3.7- and 2.4-fold, respectively. Meanwhile, the HEG and HEX outcomes improved by approximately 4.6- and 3.7-fold, respectively. Moreover, the GR yield in this experiment (42.4%) was slightly higher than that in a *V. pusilla* sample treated with H_3_PO_4_ (40.8%) [6].

### 4.5. Effect of Pretreatment on Crystalline Structure

NaOH pretreatment not only effectively solubilized lignin and partial hemicellulose from the lignin carbohydrate complex but also altered the crystallinity, porosity, and surface area of cellulose in the treated sample [42,50,51], which was also demonstrated in the present study. All treated and untreated samples maintained XRD patterns that were acceptable for cellulose I crystallinity. This implies that modifying the concentration of NaOH and temperature during sample pretreatment did not transform cellulose I into cellulose II but increased the relative *CrI* of the treated materials. NaOH-aided thermal pretreatment at 75 to 125 °C [14] diminished amorphous components (glucan, xylan, arabinan, and lignin), enhancing the proportion of relative crystalline cellulose in pretreated solids, leading to a greater *CrI* value [19,38,47,52,56,57,58,59,60].

Compared with the raw materials, the GR, HEG, XR, and HEX yields of each pretreatment condition were enhanced, which clearly indicates an increase in the relative *CrI* value but did not affect the enhanced enzymatic hydrolysis yield. NaOH pretreatment resulted in the removal of primarily amorphous components, coupled with considerable swelling and alteration within the crystalline sections of cellulose in the treated samples. These modifications improve enzymatic hydrolysis yields by rendering the cellulose surface readily available to enzymes [61,62]. These effects have been observed in various alkaline pretreatments, including wheat straw [58], *Chloris barbata* [12], *Durio zibethinus* [17], poplar [52], and sugarcane bagasse [60].

### 4.6. Effect of Pretreatment on Morphological Structure

As the original lignocellulosic feedstock has a complicated and stubborn structure, it is not readily biodegradable [14,51]. These findings suggest that changes in the surface morphology of the *V. pusilla* feedstock were significantly influenced by the NaOH concentration and temperature employed during the pretreatment. These modifications resulted from the NaOH pretreatment and temperature-dependent breaking of bonds between the lignin–polysaccharide matrix, resulting in the solubilization of hemicellulose and lignin and an increase in the porosity and surface area of cellulose [12,17,40,60,63,64,65]. Notably, adaptations enhance the efficiency of enzymatic hydrolysis by facilitating the accessibility of carbohydrates to the treated samples [25,26,64,66].

### 4.7. Ethanol Fermentation

Bioethanol can be produced from lignocellulose by the fermentation of either hexose or pentose sugars, depending on the microorganism strain. The inability of *S. cerevisiae* TISTR 5339 to convert sugar from pentose to ethanol is a limiting factor in the generation of biofuels from biomass [67]. In contrast with the standard medium, non-detoxified BH medium significantly affected yeast cell proliferation (Figure 12). Therefore, the BH medium might be contaminated with inhibitory compounds, such as phenolic compounds and aliphatic acids, which were generated during the alkaline pretreatment of the raw material preparation [52,68,69,70]. These inhibitors suppress *S. cerevisiae* TISTR 5339 growths but have no impact on bioethanol production [6,7,32,71,72]. In the present study, the fermentation medium contained both glucose and xylose. When the amount of glucose is inadequate, xylose is consumed as the carbon source, resulting in a decrease in xylose [73,74]. Cellulosic ethanol was successfully produced using *S. cerevisiae* TISTR 5339 in a non-detoxified BH medium derived from enzymatic saccharification. The maximal ethanol yield of the BH medium 89.6 ± 0.1% or 9.4 ± 0.0 g/L and the standard medium 91.8 ± 1.6% or 9.7 ± 0.2 g/L did not differ significantly (Figure 11).

Interestingly, the liquid hydrolysate, a byproduct of ethanol production, still contained a substantial quantity of xylose (7.1 ± 0.5 g/L), indicating the potential use of xylose as a substrate to produce other useful substances, such as xylitol.

### 4.8. Xylitol Production

A sustainable and cost-effective bioconversion process is essential for the successful implementation of biorefineries [75]. Therefore, xylose in the liquid hydrolysate was used as a substrate for xylitol production by *C. tropicalis* TISTR 5171.

Notably, the NaOH pretreatment of lignocellulosic materials leads to delignification, resulting in the production of lignin derivatives, particularly phenolic compounds, and aliphatic acids [52,68,69,70]. These chemical compounds effectively inhibit yeast cell proliferation and result in decreased xylitol production [69,76,77]. The potential impact of the inhibitory compounds may not be identifiable in our study because *C. tropicalis* TISTR 5171 grew better in XBH medium than in XC medium. According to these findings, both XC and XBH media were successful in producing xylitol levels higher than 50%. Nevertheless, the xylitol yield of XC medium was slightly higher than that of XBH medium. Nonetheless, the entire xylose present in both XC and XBH media was completely used. This could be attributed to the fact that yeast cells metabolize most of the xylose in the medium, leading to an inadequate amount of xylose for xylitol production. In addition, some studies have found that xylitol output and productivity rates are constrained by low xylose concentrations [76,78,79].

### 4.9. Material Balance

**Figure 15:**
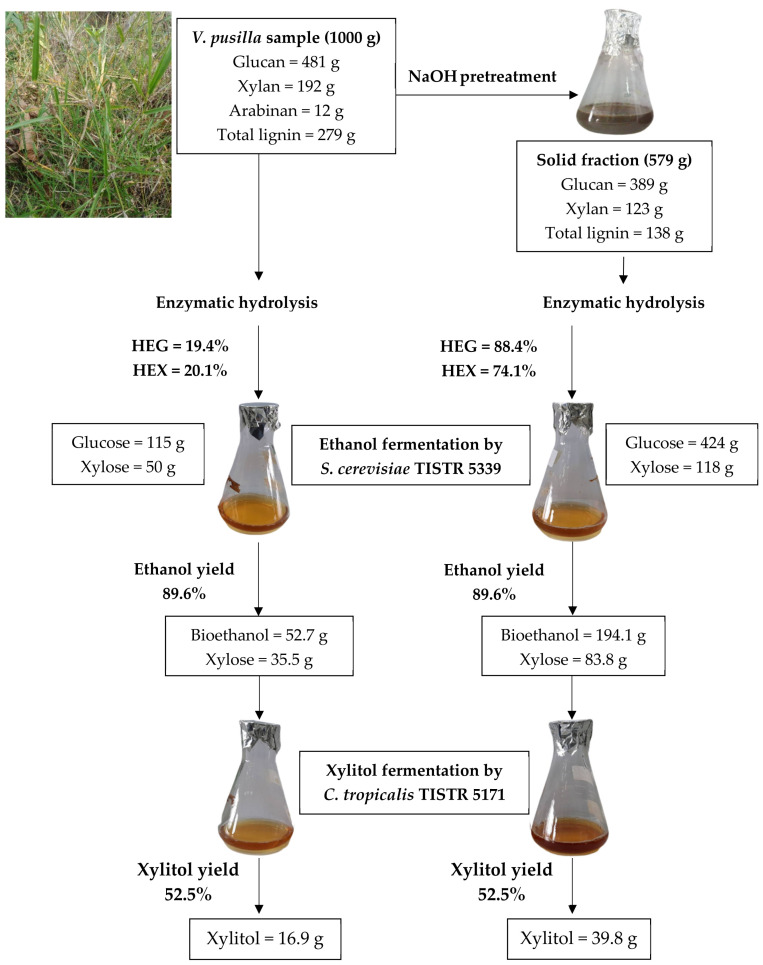
Demonstrates material balance of *V. pusilla* biomass utilized in the fermentation of bioethanol and xylitol.

Material balance calculations were conducted under the optimal NaOH-catalyzed steam pretreatment conditions to generate fermentable sugars from *V. pusilla* biomass (Figure 15). The overall process and distribution of the main components of *V. pusilla* were also evaluated. The main components of the raw material were glucan, xylan, arabinan, and total lignin. After pretreatment with 2% NaOH at 120 °C for 60 min, the mass balance indicated approximately 57.9% recovery of the pretreated material comprising 38.9% glucan, 12.3% xylan, and 13.8% total lignin. To evaluate the influence of pretreatment on enzymatic hydrolysis, untreated and pretreated samples were treated with cellulase, β-glucosidase, and xylanase. The results indicate a significant (*p* < 0.05) enhancement in GR, HEG, XR, and HEX yields in the pretreated biomass. After 96 h of hydrolysis, the pretreated biomass had an HEG of 88.4% (GR = 424 g) and an HEX of 74.1% (XR = 118 g). In contrast, the untreated sample displayed an HEG of 19.4% (GR = 115 g) and an HEX of 20.1% (XR = 50 g). The yields of GR and XR increased by approximately 3.7 and 2.4 times, respectively, compared with those of the raw material. Furthermore, there was an enhancement of around 4.6 and 3.7 times in the yields of HEG and HEX, respectively.

Subsequently, the BH obtained through enzymatic hydrolysis was used as a carbon source for bioethanol production by *S. cerevisiae* TISTR 5339. The results indicating a higher bioethanol yield from the pretreated biomass (194.1 g) than that from the untreated sample (52.7 g) provide evidence for the improvements through pretreatment and demonstrate the potential of non-detoxified BH as a carbon source for cellulosic ethanol production. Following ethanol production, the liquid hydrolysate, which consisted of xylose, was used as a carbon substrate for xylitol biosynthesis by *C. tropicalis* TISTR 5171. Notably, the xylitol output from the processed biomass (39.8 g) was roughly 2.4 times higher than that from the untreated sample (16.9 g).

Overall, we have demonstrated that the glucose and xylose in the BH and XBH medium, without any other detoxification processes, could be utilized as sustainable six- and five-carbon sources for the production of bioethanol and xylitol from *V. pusilla* feedstock, respectively. The results of this study provide vital details for the thermal pretreatment of *V. pusilla* biomass with NaOH, which is necessary for the complete utilization of six- and five-carbon sugars from weed biomass to produce two value-added products (bioethanol and xylitol). To optimize the recovery of solids, enhance the hydrolysis efficiency of glucose and xylose, and improve the recovery yields of both sugars, the influence of various pretreatment parameters, including temperature, NaOH degree, and period, was assessed using response surface methodology (RSM). However, bioethanol production can be improved by utilizing yeast strains that are tolerant to inhibitors or by eliminating inhibitors from BH before employing it in ethanol fermentation. In addition, microorganisms can become more tolerant of high ethanol concentrations through strain modifications.

To improve xylitol conversion yield, selecting microbial strains or genetically modified microorganisms that can convert xylose to xylitol with greater efficacy than natural microbial strains is crucial. Additionally, the xylitol production medium needs to be optimized. The implementation of this approach is expected to optimize the efficiency of cellulosic ethanol and xylitol production using plant-based biomass. The results highlight the potential of this simple process for producing bioethanol and xylitol, promoting the sustainable utilization of *V. pusilla* biomass, and supporting the development of a circular bioeconomy.

## 5. Conclusions

In the current study, an autoclave was employed to perform the thermal pretreatment of *V. pusilla* biomass at varying concentrations of NaOH and temperatures. The synergistic action of temperature and NaOH resulted in the breakdown of the stiff structure of *V. pusilla* biomass, resulting in delignification, an increase in cellulose porosity and surface area, and enhanced enzymatic hydrolysis yield. *V. pusilla* feedstock was efficiently pretreated with 2% NaOH at 120 °C for 60 min. Under these circumstances, the most outstanding yields of glucose recovery (42.4 ± 0.0%) and glucose hydrolysis efficiency (88.4 ± 0.0%) were achieved. However, delignification was only 50.5 ± 0.1%, indicating that complete lignin removal is not mandatory. In addition, glucose and xylose in the BH and XBH media, respectively, could potentially be utilized as renewable hexose and pentose carbon sources to generate bioethanol and xylitol from *V. pusilla* feedstock without any additional detoxification operations. The yields of bioethanol (194.1 g) and xylitol (39.8 g) were considerably higher than those obtained from untreated biomass. Therefore, *V. pusilla* biomass has a great potential for application in sugar platform-based biorefineries for the production of bioethanol, xylitol, and other high-value products. Nevertheless, to optimize the recovery yields of solids, glucan, xylan, hydrolysis efficiency, and the products of glucose and xylose recoveries, the pretreatment variables temperature, NaOH concentration, and duration were evaluated using statistical techniques such as RSM. Enhancing xylitol output requires improving the microbial strains used to convert xylose more efficiently into xylitol. Optimizations of the medium utilized for xylitol synthesis is also recommended.

## Figures and Tables

**Table 1:** Chemical compositions of *V. pusilla* after NaOH pretreatment at 120 °C.

Composition (% DW)	Untreated Sample	Concentration of NaOH (%)			
		1	2	3	4
Glucan	48.1 ± 0.3 ^e^	55.3 ± 0.5 ^d^	67.2 ± 0.1 ^c^	68.0 ± 0.0 ^b^	71.0 ± 0.2 ^a^
Xylan	19.2 ± 0.4 ^c^	21.9 ± 0.2 ^a^	21.3 ± 0.3 ^a^	20.1 ± 0.2 ^b^	17.8 ± 0.4 ^d^
Arabinan	1.2 ± 0.1	n.d.	n.d.	n.d.	n.d.
AIL	23.5 ± 0.1 ^a^	21.8 ± 0.0 ^b^	20.9 ± 0.1 ^c^	18.1 ± 0.5 ^d^	15.4 ± 0.3 ^e^
ASL	4.4 ± 0.1 ^a^	3.0 ± 0.0 ^b^	2.9 ± 0.0 ^b^	2.9 ± 0.0 ^b^	2.9 ± 0.0 ^b^
Total lignin	27.9 ± 0.2 ^a^	24.8 ± 0.1 ^b^	23.9 ± 0.1 ^c^	21.0 ± 0.5 ^d^	18.3 ± 0.3 ^e^

The superscripted characters within rows indicate the statistical significance of the differences between them (*p* < 0.05), n.d. = not determined.

**Table 2:** Chemical compositions of *V. pusilla* after 2% NaOH pretreatment at different temperatures.

Composition (% DW)	Untreated Sample	Temperatures (°C)		
		110	120	130
Glucan	48.1 ± 0.3 ^c^	65.1 ± 0.3 ^b^	67.2 ± 0.1 ^a^	67.1 ± 0.3 ^a^
Xylan	19.2 ± 0.4 ^c^	22.5 ± 0.1 ^a^	21.3 ± 0.3 ^b^	19.3 ± 0.2 ^c^
Arabinan	1.2 ± 0.1	n.d.	n.d.	n.d.
AIL	23.5 ± 0.1 ^a^	23.0 ± 0.1 ^a^	20.9 ± 0.1 ^b^	16.1 ± 0.5 ^c^
ASL	4.4 ± 0.1 ^a^	3.2 ± 0.0 ^b^	2.9 ± 0.0 ^c^	2.9 ± 0.0 ^c^
Total lignin	27.9 ± 0.2 ^a^	26.1 ± 0.1 ^b^	23.9 ± 0.1 ^c^	19.0 ± 0.5 ^d^

The superscripted characters within rows indicate the statistical significance of the differences between them (*p* < 0.05), n.d. = not determined.

**Table 3:** The crystallinity index (*CrI*) of the untreated and NaOH-pretreated of *V. pusilla.*

Concentration (%)	Temperature (°C)	*CrI* (%)
Untreated	-	59.5
1% NaOH	120	66.2
2% NaOH	120	67.6
3% NaOH	120	67.7
4% NaOH	120	67.9
2% NaOH	110	68.5
2% NaOH	130	67.4

## Data Availability

Not applicable.
